# Dendritic Cells and CD8 T Cell Immunity in Tumor Microenvironment

**DOI:** 10.3389/fimmu.2018.03059

**Published:** 2018-12-20

**Authors:** Chunmei Fu, Aimin Jiang

**Affiliations:** Department of Immunology, Roswell Park Comprehensive Cancer Center, Buffalo, NY, United States

**Keywords:** CD103^+^ cDC1s, CD8 T cell immunity, anti-tumor immunity, cross-priming, tumor microenvironment, cancer immunotherapy

## Abstract

Dendritic cells (DCs) play a central role in the regulation of the balance between CD8 T cell immunity vs. tolerance to tumor antigens. Cross-priming, a process which DCs activate CD8 T cells by cross-presenting exogenous antigens, plays a critical role in generating anti-tumor CD8 T cell immunity. However, there are compelling evidences now that the tumor microenvironment (TME)-mediated suppression and modulation of tumor-infiltrated DCs (TIDCs) impair their function in initiating potent anti-tumor immunity and even promote tumor progression. Thus, DC-mediated cross-presentation of tumor antigens in tumor-bearing hosts often induces T cell tolerance instead of immunity. As tumor-induced immunosuppression remains one of the major hurdles for cancer immunotherapy, understanding how DCs regulate anti-tumor CD8 T cell immunity in particular within TME has been under intensive investigation. Recent reports on the Batf3-dependent type 1 conventional DCs (cDC1s) in anti-tumor immunity have greatly advanced our understanding on the interplay of DCs and CD8 T cells in the TME, highlighted by the critical role of CD103^+^ cDC1s in the cross-priming of tumor antigen-specific CD8 T cells. In this review, we will discuss recent advances in anti-tumor CD8 T cell cross-priming by CD103^+^ cDC1s in TME, and share perspective on future directions including therapeutic applications and memory CD8 T cell responses.

## Introduction

Cancer is characterized by the accumulation of genetic mutations and the loss of normal cellular regulatory functions (1). The identification of tumor-associated antigens (TAAs) that separated cancerous cells from non-transformed healthy cells, and the observation of tumor antigen-specific CD8 T cells in cancer patients have greatly advanced our understanding on tumor immunology and formed the basis for antigen-specific immunotherapy (2). The first human tumor antigen recognized by CD8 cytotoxic T lymphocytes (CTL) was identified in melanoma and was designated melanoma-associated antigen (MAGE)-1 (3). The isolation of tumor-specific CTL from peripheral blood or tumor tissue of patients from various cancer patients provided evidence for existence of CD8 T cell-mediated anti-tumor immunity (4–7). The detection of TAA-specific CD8 T cells in spontaneously regressing tumors further supported the importance of tumor-specific CD8 T cell responses (5). It is well accepted now that CD8 T cells play a central role in mediating anti-tumor immunity, and their effector CTLs eliminate tumor cells by recognizing tumor-associated antigens presented on major histocompatibility complex class I (MHCI) by their expressed T cell receptor (TCR). Indeed, studies have shown that infiltration of T cells, especially CD8 T cells into tumor microenvironment, correlates with better prognosis in multiple malignancies such as breast, lung, melanoma, colorectal, and brain cancer (8, 9). However, even when tumor-specific CD8 T cell responses were observed, they rarely provided protective immunity as tumors often evade immune surveillance by dampening T cell effector and memory functions (10, 11). Promising cancer immunotherapies that aim to boost CD8 T cell-mediated anti-tumor immunity include DC cancer vaccines, adaptive cell transfer (ACT) of tumor-reactive T cells, either native (CTL clones or Tumor infiltrated lymphocytes–TIL) or engineered to express tumor antigen-specific TCR or chimeric antigen receptors (CAR), and immune checkpoint blockade (ICB) such as anti-PD-1, anti-PD-L1, and anti-CTLA-4 (2). Among them, immunotherapies with ICB and CAR T cells have achieved unprecedented clinical efficacy leading to a number of drugs being approved by the FDA. However, a majority of patients still fail to respond to these checkpoint or CAR T cell therapies, and many patients that do respond often experience relapse (12). While direct presentation of tumor antigens onto their MHCI by tumor cells play an important role in effector function of CD8 T cells, cross-presentation by professional antigen presenting cells in particular DCs are required for prime naive CD8 T cells and sustaining the cytotoxic immune responses (13). Thus, increasing efforts has been made to repair and enhance insufficient T cell priming by DCs to further improve the efficacy of immunotherapies with ICB and CAR T cells due to DCs' critical role in priming and directing CD8 T cells to target tumor cells (12, 14). Indeed, the ability of DCs to cross-present exogenous tumor-associated antigens onto MHCI molecule to prime CD8 T cells is the foundation of the “Cancer-Immunity cycle” proposed by Chen and Mellman (11). Thus, better understanding the interaction of CD8 T cells and DCs would be critical to improve the efficacy of current cancer immunotherapies.

## Dendritic Cells and Tumor Microenvironment

Ralph Steinman was awarded the 2011 Nobel Prize for Medicine or Physiology for his pioneering work on DCs (15). As the sentinel of the immune system, DCs play a central role in linking innate and adaptive immune responses (16). Known as the most potent professional antigen presenting cells (APCs), DCs initiate all adaptive immune responses by uptaking, processing and presenting antigens including tumor antigens to activate naive antigen-specific CD4 and CD8 T cells (17). Since their identification in 1973 (18), DC development and the regulation of their function have been under intensive study. DCs originate in bone marrow from macrophage/DC progenitors (MDP) that give rise to common DC progenitors (CDP), which then differentiate into two major DC subsets: classical/conventional DCs (cDCs) and plasmacytoid DCs (pDCs) (19–26). Murine cDCs consist of two subtypes currently described as cDC1s (XCR1^hi^CD24^hi^CD26^hi^CD11c^hi^MHCII^hi^ CD11b^lo^CD172a^lo^F4/80^lo^CD64^lo^Lin^lo^,type 1 cDCs)andcDC2s(CD11b^hi^CD172a^hi^CD26^hi^CD11c^hi^MHCII^hi^XCR1^lo^F4/80^lo^CD64^lo^Lin^lo^, type 2 cDCs), and their human counterparts are CD141^+^ DCs (also known as BDCA3^+^) and CD1c^+^ DCs (also known as BDCA1^+^), respectively (27, 28). These two subtypes of cDCs differ in their transcriptional factor dependency, function and phenotypes (23, 24). cDC1 cells include lymphoid tissue CD8α^+^ cDC1s and migratory CD103^+^ cDC1s (29). cDC1 cells rely on interferon regulatory factor 8 (IRF8) and basic leucine zipper transcriptional factor ATF-like 3 (Batf3) for their development, and are specialized in presenting internalized exogenous antigens onto MHCI to prime CD8 T cells by cross-presentation (30). cDC2s depend on interferon regulatory factor 4 (IRF4) for their development and comprise a heterogeneous population that are very efficient in presenting internalized antigens on MHCII to activate CD4 T cells (31–34).

pDCs are a multifunctional population best known for their specialized ability in producing and secreting large amount of type I interferons (IFNs) (35–37). pDCs also express high level of IRF8 similar to cDC1s, but require the E2-2 transcription factor for their development (38). E2-2, encoded by TCF4, is a member of the E family of basic helix–loop–helix transcription factors (39). In both mice and humans, E2-2 is required for the differentiation of pDCs from CDPs (38). Induced deletion of E2-2 in mature pDCs results in the acquisition of cDC-like properties, such as dendritic morphology, MHCII and CD8α expression, and the ability to induce proliferation of allogeneic CD4 T cells (40). Deletion of E2-2 in pDCs also induces the expression of ID2, which is required for cDC1 development. Murine pDCs express Siglec-H, B220, Ly6c, PDCA1 (CD317) and intermediate level of CD11c, and human pDCs express HLA-DR, CD123, BDCA2 (CD303), and BDCA4 (CD304) but not CD11c (36, 41). Initially reported as IFN-producing cells (IPCs), pDCs have been extensively studied for their function in sensing viral RNA and DNA by toll-like receptor (TLR)-7 and−9 (42, 43). In addition to their function in producing IFNs, pDCs have also been shown to play an important role in immune tolerance. In autoimmune diseases, aberrant activation of pDCs has been implicated in the pathogenesis of psoriasis, systemic lupus erythematosus (SLE), and IFN-related autoimmune diseases (36, 44, 45).

Monocytes that arise from MDPs could also differentiate into another DC subset named Monocyte-derived inflammatory DCs (inf-DC) under conditions such as inflammation, cancer and infection (46). The inf-DCs have been shown to both activate antigen-specific CD4 T cells and cross-present tumor antigens to activate CD8 T cells, and their presence has been found to be important for the efficacy of cancer immunotherapy (47–49). Recently, TNF/iNOS-producing DCs (TIP-DCs), a novel type of inf-DCs that produce TNF-α and nitric oxide (NO) was shown to be critical for tumor growth control upon treatment with adaptive CD8 T cell transfer (50).

The TME is a specialized niche composed of tumor cells, fibroblasts, endothelial cells, infiltrating leukocytes, and extracellular matrix components. TIDCs have been found in many cancer types including breast, lung, renal, head and neck, gastric, colorectal, bladder and ovarian cancers (51). However, in general within the TME tumor cells are able to adapt their environment to favor tumor growth, evade immune surveillance and confer resistance to immunotherapies (52, 53). A key mechanism in achieving tumor immune evasion is through modulation of DC function by tumors and tumor-associated cells/factors in the TME. Thus, despite the presence of DCs in TME and their potential in generating anti-tumor immunity, TIDCs often exhibit impaired or defective function, thus might mediate immunosuppression instead (41, 54). Indeed, the TME employs a variety of mechanisms to modulate DCs to suppress their ability to induce anti-tumor responses.

## DC-Suppressive Molecules in TME

A number of factors such as IL-6, Macrophage colony-stimulating factor (M-CSF), IL-10, Vascular endothelial growth factor (VEGF), and Transforming growth factor beta (TGF-β) that are present in TME have been shown to negatively regulate DC functions (55, 56). IL-6 and M-CSF, cytokines secreted by tumor cells, have been shown to switch the differentiation of CD34^+^ progenitors from DCs to CD14^+^ monocytes that failed to mediate allogeneic T cell proliferation (57, 58). Tumor-derived IL-6 has been shown to negatively regulate DC function by inhibiting their maturation and migration, affect the differentiation of hematopoietic progenitor cells from DCs to macrophage, and induce tolerogenic phenotypes of DCs (59–61). In the TME, a variety of cells such as tumor cells, myeloid-derived suppressor cells (MDSCs), tumor-associated macrophages (TAMs), DCs, and Tregs, have been shown to produce IL-10 (62). IL-10 has been shown to suppress DC function by inhibiting different aspects of DC biology, such as DC maturation, their ability to secret IL-12, their capacity in antigen presentation and priming of T cells (63, 64). IL-10 has also been shown to convert immunogenic DCs into tolerogenic DCs leading to the induction of anergic cytotoxic CD8 T cells (65). In addition, IL-10 derived from tumors has also been shown to switch differentiation from monocytic precursors to immunosuppressive TAMs rather than DCs (66). VEGF has been shown to inhibit differentiation and maturation of DCs (67, 68). Tumor-derived TGF-β significantly suppresses DC function and their ability to initiate anti-tumor immune responses by inhibiting DC maturation (69, 70).

Several factors such as VEGF, TGFβ, IL-1β, IL-13, Granulocyte-macrophage colony-stimulating factor (GM-CSF) and prostaglandins, that are produced by tumor cells and other cells in the TME, have been shown to inhibit DC differentiation from progenitors and promote their differentiation into immunosuppressive cells such as MDSCs and TAMs (71).

Another mechanism used by TME to evade immune detection is by modulating DC function to skew T cell differentiation. Factors in TME, such as Matrix metalloproteinase 2 (MMP-2) and Thymic Stromal Lymphopoietin (TSLP) have been shown to modulate DC function to induce detrimental Th2 responses (72, 73). Tumor-produced TSLP has been shown to up-regulate OX40L expression on DCs, thus inducing the generation of Th2 cells that produce IL-4 and IL-13 that have been shown to promote tumor growth in breast and pancreatic cancer (74, 75).

Several signaling pathways such as β-catenin, MAPK and STATs that are active in cancers also play critical roles in crosstalk between tumor cells and DCs in the TME (76, 77). β-catenin signaling in melanoma cells has been shown to inhibit the recruitment of T cells and DCs into tumors (78). Melanoma-derived Wnt ligand Wnt5α has been shown to increase the production of Indoleamine 2,3-dioxygenase (IDO) by TIDCs via β-catenin signaling, leading to increased generation of T_reg_ cells (79). Conditional knockout of Wnt co-receptors LRP5 and LRP6 on DCs, on the other hand, enhanced DC-mediated anti-tumor immunity leading to delayed tumor growth (80). In addition, activation of β-catenin in DCs from tumor-bearing mice exhibited a more tolerogenic phenotype and mediated the suppression of DC vaccine-induced cross-priming of anti-tumor CD8 T cells through IL-10 (81, 82).

## Regulatory T Cells

Regulatory T cells (Tregs), working in concert with tolerogenic DCs, play critical roles in the establishment and maintenance of an immunosuppressive TME to inhibit anti-tumor immunity (83). Tregs are comprised of a heterogeneous population of T lymphocytes that have shared the ability to suppress immune responses, with the CD4^+^CD25^+^Foxp3^+^ Tregs being most studied. These Tregs express the inhibitory receptors CTLA-4, Tim-3, PD-1, GITR, LAG3, and BTLA that exert their suppressive function on DCs through different mechanisms. For example, Tregs have been shown to inhibit DC maturation by down-regulating the expression of co-stimulatory molecules such as CD80 and CD86 through CTLA-4 (84). Engagement of CTLA-4 on Treg by CD80/CD86 on DCs has been shown to up-regulate both human and murine DCs' production of IDO (85), which then activate antigen-specific regulatory T cells to induce potent suppressor activity (86, 87). In turn, IDO-activated Tregs have been shown to induce the up-regulation of the inhibitory PD-L1 on DCs (88). In addition, Tregs secrete IL-10 and TGF-β, two of the main immunosuppressive cytokines that are known to induce DC dysfunction (89, 90).

## Expression of Inhibitory Ligands

The expression of inhibitory molecules, such as PD-L1, PD-L2, Tim3, LAG3 contributes to the suppressed function of DCs in tumors and tumor-draining LNs. It has been reported that tumor-derived factors up-regulate Tim3 expression in tumor DCs (91). TIM-3 on DCs then inhibits anti-tumor responses and reduces the efficacy of cancer treatments by binding to high-mobility group box 1 protein (HMGB1), a damage-associated molecular pattern molecule involved in cytosolic nucleic acid recognition in the TME. In addition, signaling via TIM-3 on both BMDCs and splenic DCs has been shown to inhibit DC activation and maturation (92). For PD-L1, CD103^+^ DCs from tumor-draining LNs have recently been shown to have increased expression of PD-L1 compared to non-draining LN DCs (93). PD-L1 and PD-1 blockade has been shown to reverse DC dysfunction leading to enhanced T cell immunity (94, 95), suggesting that PD-L1/PD-1 signaling negatively regulates DC function.

## Inhibition of Antigen Presentation Function of TIDCs

Tumor cells escape immune surveillance by disabling the process of tumor antigen presentation. Recent studies have shown that DCs in TME often exhibited impaired capacity in cross-presentation (25, 96). The TME can specifically modulate DCs' antigen presentation function by targeting the molecules and machinery directly involved in antigen presentation, for example, decreasing the expression of their MHCI and MHCII molecules and their regulators such as CIITA, down-regulation of genes such as ER-resident aminopeptidases (ERAP) and transporter associated with antigen processing (TAP) (97). Abnormal accumulation of lipids in DCs has emerged as an important mechanism for DC dysfunction, as TIDCs from multiple tumor models and cancer patients exhibited reduced capacity in cross-presentation because of lipid accumulation (98, 99). Supporting this notion, a recent study has shown that the accumulation of lipids in TIDCs was involved inplay arole in blunting inhibiting anti-tumor T cell responses in ovarian cancer (100).

## pDCs in TME

Recruitment of pDCs to the tumor microenvironment has been reported in a variety of cancers, however, these tumor-infiltrated pDCs are often tolerogenic, favoring tumor progression. High tumor infiltration by pDCs has been associated with poor prognosis in melanoma, head and neck, breast, and ovarian cancers (45, 101–103). pDCs have been shown to induce the generation of Tregs in the TME and tumor-draining LNs (88, 104). pDCs can also stimulate the generation of Tregs by their expression of ICOS-L, and ICOS-L expression on pDCs has also been shown to be associated with breast cancer progression (105, 106). On the other hand, pDCs have also been shown to promote immunogenic anti-tumor responses if properly stimulated, as therapeutic activation of pDCs have shown efficacy in melanoma, basal cell carcinoma, and T cell lymphoma (25, 41, 103, 107).

## Dendritic Cells in Cross-Priming of Anti-Tumor CD8 T Cells and Beyond

Cross-priming, a process which DCs activate CD8 T cells by cross-presenting exogenous antigens (108, 109), plays a critical role in generating anti-tumor CD8 T cell immunity (110–115). Anti-tumor CD8 T cell responses are induced in three sequential steps: (1) tumor antigen uptake and cross-presentation; (2) tumor antigen-specific CD8 T cell priming by DCs, and (3) elimination of tumor cells by effector CTLs (116). However, TME-mediated suppression and modulation of TIDCs often leads to their dysfunction, resulting in failure in cross-priming (step 1 and 2) and suppressed anti-tumor CD8 T cell immunity. Indeed, DC-mediated cross-presentation of tumor antigens in tumor-bearing hosts often induces T cell tolerance instead of immunity (110). However, not all TIDCs within TME exhibit suppressive and/or regulatory functions. For example, the infiltration of BDCA3^+^ cDC1s in the TME has been shown to correlate with increased T cell infiltration and improved prognosis in cancer patients and better efficacy of cancer immunotherapies, highlighting the critical positive role of cDC1 in generating anti-tumor immunity in the TME (78, 117). Thus, recent discoveries on the critical role of cDC1s in particular CD103^+^ cDC1s in CD8 T cell cross-priming in tumors have generated much interest, and have offered opportunities for improved cancer immunotherapies (96).

The generation of Batf3^−/−^ mice that selectively lack cDC1s has greatly advanced our understanding of their function in CD8 T cell cross-priming in tumors (30). Batf3^−/−^ mice exhibited defective cross-presentation and impaired anti-tumor immunity, suggesting that cDC1s play a critical role in initiating CD8 T cell-mediated anti-tumor immunity through cross-presentation (30). The mechanisms that make cDC1s superior in cross-presentation are only being uncovered recently. While cDC1s exhibit high efficiency at endocytosis of cell-associated antigens, their superior capacity in cross-presentation is thought to due to their specialized capability in processing antigens (96). In addition, the cross-presentation capacity of cDC1s is further enhanced by their expression of Clec9A, which facilitate the cross-presentation of antigens from dead cells by binding filamentous actin (118–120).

**Figure 1:**
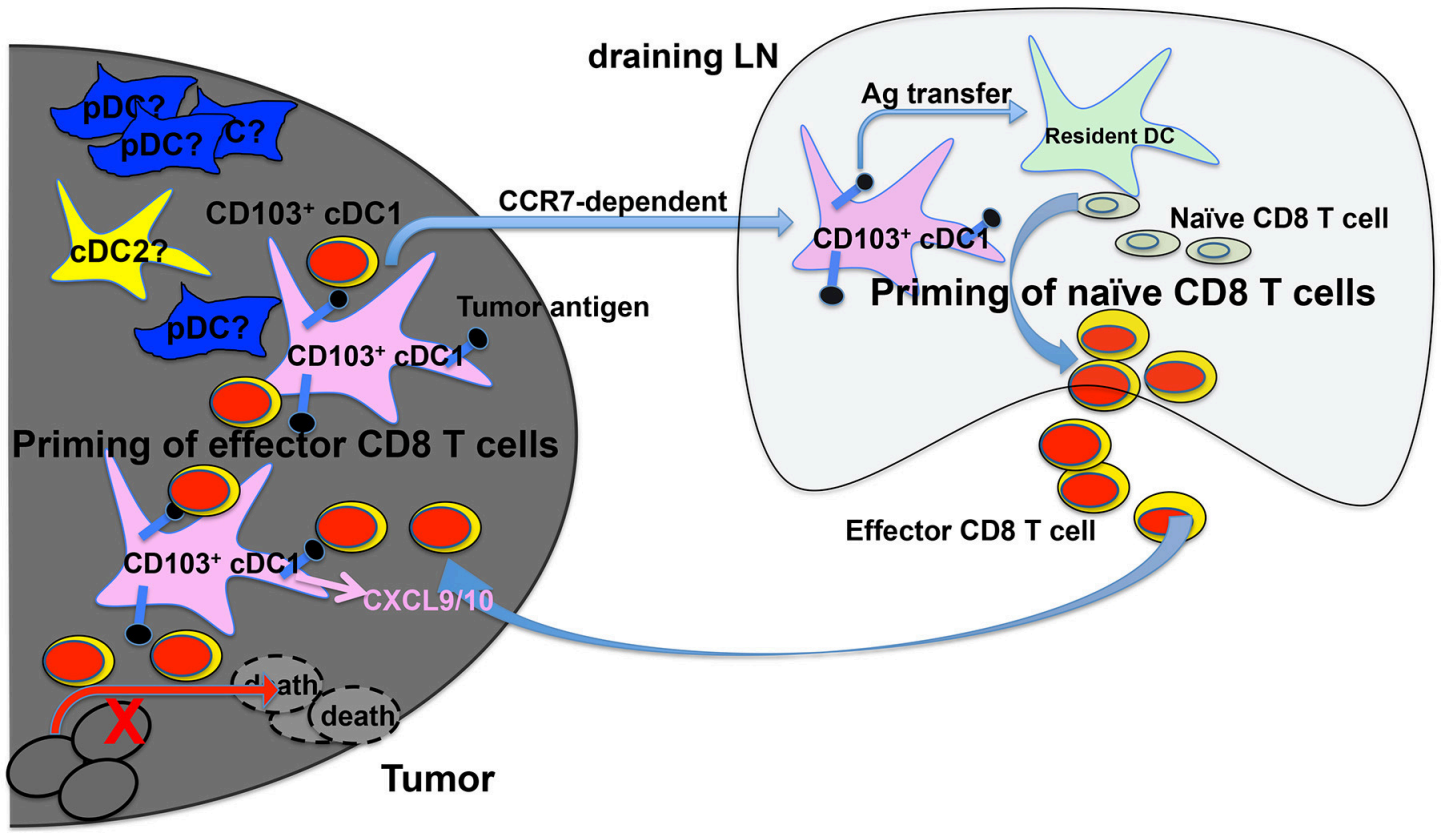
cDC1s and priming of tumor-antigen-specific CD8 T cells in the tumor microenvironment (TME) and tumor-draining lymph nodes (tdLNs). Migratory CD103^+^ cDC1s in the TME take up tumor antigens (black dots), and transport tumor antigens to tdLN by migrating to the tdLN in a CCR7-dependent mechanism. Once in the tdLN, cross-presenting CD103^+^ cDC1s prime naive tumor antigen-specific CD8 T cells to become effector CD8 T cells. Cross-presenting CD103^+^ cDC1s also transfer tumor antigens to other resident myeloid cells including CD8α^+^ cDC1s that are also likely involved in priming naive CD8 T cells in tdLN. cDC1s in the TME produce CXCL9/10 to recruit primed effector CD8 T cells into TME, where they are re-stimulated by CD103^+^ cDC1s leading to the efficient killing of tumor cells. The function of other DCs such as pDCs and cDC2s in CD8 T cell priming is less understood.

Examining the TME, Broz et al. have identified CD103^+^ cDC1s as the only population with the capability to induce proliferation of both naive CD8 T cells and established CTLs, suggesting that CD103^+^ cDC1s are the APCs that cross-prime CTLs in the TME (117) (Figure 1). More importantly, analysis of The Cancer Genome Atlas (TCGA) database indicated that the CD103^+^/CD103^−^ gene ratio correlates strongly with increased patient survival across 12 different cancer types (117). Consistently with cDC1s' critical role in anti-tumor immunity, a recent study has shown that activation of β-catenin signaling in melanoma cells reduces the numbers of intratumoral CD103^+^ cDC1 cells, thus preventing tumor-specific T cell priming, suggesting that CD103^+^ cDC1s might not only promote anti-tumor immunity but also be suppressed by cancer cells for immune evasion (78). In both B16 and Braf-mutant mouse melanoma models, CD103^+^ cDC1s have been shown to play a critical role in the efficacy of immunotherapy with PD-L1 and Braf inhibition (93). A combined treatment of systemic FMS-like tyrosine kinase 3 ligand (FLT3L) and poly I:C at the tumor sites, which induced the expansion and maturation of CD103^+^ cDC1s, improved the efficacy of BRAF and PD-L1 blockade, suggesting that combined FLT3L and poly I:C therapy might be a promising approach that could improve the efficacy of current ICB immunotherapy in cancer patients (93). Similarly, efficacy of immunotherapy using PD-1 and CD137 blockade has been shown to depend on CD103^+^ cDC1s, likely due to their function in cross-priming (121).

Recent studies have also shown that CD103^+^ cDC1s are the only population that mediates the transport of solid tumor antigens from TME to tumor draining lymph nodes for cross-priming of CD8 T cells (93, 122) (Figure 1). DC migration to LNs is mediated by the CCR7 chemokine receptor expressed on CD103^+^ cDC1s, as CCR7^−/−^ CD103^+^ cDC1s exhibited reduced function in migration and T cell priming (122). In addition, intratumoral CD103^+^ cDC1s also play a critical role in the trafficking of tumor-specific effector T cells into tumors, as effector T cell recruitment into tumors depends on the presence of CXCL9/10-producing CD103^+^ cDC1s (123, 124) (Figure 1). Importantly, the expression of Batf3-dependent DC transcripts in human melanoma tumors correlates with CXCL9/10 expression and CD8 T cell infiltration, suggesting that Batf3-dependent cDC1s might regulate T cell recruitment to tumors in both mice and human (124).

The role of cDC2s and pDCs in CD8 T cell cross-priming in tumors are less well understood. cDC2s isolated from the TME have been shown to engulf tumor antigens and induce T cell proliferation *in vitro*, suggesting cDC2s may play a role in cross-priming CD8 T cells in TME (117). Given the dominant role of cDC1s as described above, however, cDC2s likely play a minor role in promoting anti-tumor immunity. pDCs can present antigens to activate CD4 T cells as well as activate CD8 T cells through cross-presentation (125, 126). Recruitment of pDCs to the TME has been reported in a number of cancers, although high tumor infiltration of pDCs has been shown to correlate with poor prognosis in melanoma, head and neck, breast, and ovarian cancers (45, 101–103). Activation of pDCs has been shown to promote anti-tumor immunity, likely through the production of type 1 IFNs (127, 128). The role of tumor infiltrated pDCs in the cross-priming of tumor-specific CD8 T cells, however, remains underinvestigated and poorly understood. Interesting, several recent reports have shown the cooperation of pDCs and cDCs in achieving optimal cross-priming (129, 130), suggesting that pDCs could play a positive role in generating anti-tumor CD8 T cell immunity.

## Strategies Targeting DC Function in CD8 T Cell Priming to Improving the Efficacy of Cancer Immunotherapies

It's worth noting that the best efficacy for anti-CTLA-4 blockade was achieved in combination with GM-CSF^+^ tumor cell vaccination two decades ago by the lab of the newly Nobel laureate Dr. James Allison (131, 132). In the 1998 PNAS paper, the authors suggested that “the most effective and synergistic vaccine strategy targets treatments that enhance T cell priming at the level of host-derived antigen-presenting cells” (131), which quite accurately predicted the direction of cancer immunotherapy as combining ICB with DC-based cancer immunotherapy. In light of the recent discovery of the critical role of cDC1s in priming tumor-specific CD8 T cells, repairing and/or enhancing DC-mediated CD8 T cell priming represents an exciting approach to improve the efficacy of current T cell-based cancer immunotherapies including ICB and ACT (12, 14, 96). Indeed, Spranger et al. have shown that vaccination with *in vitro*-generated DCs improved the efficacy of anti-PD-L1 and anti-CTLA-4 immunotherapy (78). Similarly, treatment of FLT3L/poly I:C, which expands and induces the maturation and activation of CD103^+^ cDC1s at the tumor sites, has been shown to enhance anti-tumor responses and improve efficacy when combined with BRAF and PD-L1 blockade (93). Recently, we have genetically engineered tumor-specific CD8 T cells with a second T-cell receptor (TCR) that recognizes a *Listeria* antigen. And we have shown that *Listeria* infection led to the eradication of primary tumors and development of immunological memory against tumor re-challenge in combination with adoptive cell transfer (ACT) of these dual-specific T cells, likely due to the substantially enhanced T cell priming involving DCs (133). *In vivo* DC-targeted vaccines that deliver tumor antigens to cross-presenting DCs with monoclonal antibodies carrying tumor antigens is another attractive approach to enhance cross-priming of tumor-specific CD8 T cells. As multiple clinical trials with human anti-DEC-205 monoclonal antibody fused with antigens such as tumor antigen NY-ESO-1 have shown promising results (134–137), it will be interesting to combine *in vivo* DC-targeted vaccines with T cell-based cancer immunotherapies such as ICB and ACT to further improve their efficacy. Another intriguing approach is the manipulation of pDCs. While tumors are known to prevent the infiltration of cDCs exemplified by recent reports involving β-catenin signaling pathway (78), accumulation of pDCs has been reported in multiple tumors including melanoma, head and neck, breast, and ovarian cancers (45, 101–103), thus offering an opportunity to manipulate these pDCs to generate anti-tumor immunity in the tumor microenvironment (TME). Indeed, therapeutic activation of pDCs have been reported to induce immunogenic anti-tumor responses and shown efficacy in multiple human cancers (25, 41, 103, 107). While the roles of cross-priming by pDCs *in vivo* are still under debate (29, 138–140), recent studies have shown that the co-operation of pDCs and cDCs was required to achieve optimal cross-priming of CD8 T cells (129, 130, 141). Thus, studies are warrantied to further understand the contribution of other DC subsets including pDCs and cDC2s in CD8 T cell priming in TME and tumor-draining LN, which will help develop better strategies to improve efficacy of cancer immunotherapies by enhancing DC function in CD8 T cell priming.

## Memory CD8 T Cells

Generation of durable memory CD8 T cells responses that are capable of protection from recurrence and relapse is the ultimate goal of cancer immunotherapy. Memory CD8 T cells are heterogeneous populations that include both circulating memory CD8 T cells and non-circulating tissue resident memory CD8 T cells (T_rm_) (142). Circulating memory CD8 T cells can be further divided into stem cell memory (T_scm_), central memory (T_cm_) and effector memory (T_em_). Tumor infiltrated Tcm and Tem cells have been reported in multiple cancers such as colorectal and breast cancer (143–145). However, memory CD8 T cells in tumors often exhibit dysfunctional phenotypes and their dysfunction correlates with cancer progression (142). Highlighting their role in anti-tumor immunity, intratumoral expansion of T_em_ cells in patient samples have been associated with improved responses to anti-PD-L1 therapy (146). For the recently identified T_rm_ cells, tumor infiltrated CD8^+^CD103^+^ T_rm_ cells have been reported in tumor samples of ovarian, endometrial, breast and lung cancer patients, and their number correlates with prolonged survival and better prognosis (147–152). While the presence of the memory CD8 T cells in tumors is clear, whether and how TIDCs in particular CD103^+^ cDC1s regulate the generation and function of memory CD8 T cells remains largely unexplored. Under certain conditions, cross-priming of CD8 T cells by CD103^+^ cDC1s in TME does lead to memory CD8 T cell responses. For instance, Salmon et al. have shown that FLT3L/poly I:C treatment synergized with PD-L1 blockade to prevent the secondary melanoma lesions after Braf inhibition, as well as provide protection against tumor re-challenge, indicated the generation of memory CD8 T cell responses after CD8 T cell priming (93). Thus, further studies on memory CD8 T cells in TME are warrantied to understand how to better achieve memory CD8 T cell responses in TME.

## Conclusion

DC-mediated cross-priming of tumor-specific CD8 T cells plays a critical role in initiating and sustaining anti-tumor immunity (110–115). TME employs an array of mechanisms to modify the phenotype and function of TIDC to transform them into immunosuppressive DCs. Insufficient T cell priming likely contributes to cold tumors (no T cell infiltration in TME) and unresponsiveness to immune checkpoint blockade (ICB) therapy, and is under intensive investigation (12). Recently, a number of studies have shown that CD103^+^ cDC1s in TME are critical in cross-priming CD8 T cells to generate anti-tumor immunity. These CD103^+^ cDC1s mediate cross-presentation and transport tumor antigens from tumors to draining LN to prime naive CD8 T cells, have the capacity to prime tumor-reactive CTLs in TME, play a critical role in trafficking of effector CD8 T cells to tumors, thus impact all three steps of anti-tumor CD8 T cell responses required for tumor eradication (78, 93, 117, 121–124, 153). In addition, the presence of CD103^+^ cDC1s has been shown to be critical for efficacy of multiple ICB therapies (93, 121). Thus, manipulating CD8 T cell cross-priming by cDC1s, by employing strategies to increase the number of cDC1s and enhancing their capacity of cross-priming in tumors and tumor draining LNs, represents an exciting approach to enhance anti-tumor CD8 T cell immunity and improve the efficacy of current cancer immunotherapies including ICB and ACT (see reference 13 for an excellent recent review on DC-based cancer immunotherapy). Of note, combination treatment of FLT3L/poly I:C, which expands and induces the maturation and activation of CD103^+^ cDC1s at the tumor sites, has already been shown to enhance anti-tumor responses to BRAF and PD-L1 blockade (93).

## Author Contributions

All authors listed have made a substantial, direct and intellectual contribution to the work, and approved it for publication.

### Conflict of Interest Statement

The authors declare that the research was conducted in the absence of any commercial or financial relationships that could be construed as a potential conflict of interest.
